# Do Obesity-Related Traits Affect Prostate Cancer Risk through Serum Testosterone? A Mendelian Randomization Study

**DOI:** 10.3390/cancers15194884

**Published:** 2023-10-08

**Authors:** Chi Yuan, Zhongyu Jian, Shijian Feng, Menghua Wang, Liyuan Xiang, Hong Li, Xi Jin, Kunjie Wang

**Affiliations:** 1 Department of Pediatric Surgery, West China Hospital, Sichuan University, Chengdu 610041, China; chiyuan@wchscu.cn; 2 Department of Urology and Institute of Urology, Laboratory of Reconstructive Urology, West China Hospital, Sichuan University, Chengdu 610041, China; jzyhx@scu.edu.cn (Z.J.); royanniefeng@gmail.com (S.F.); 2016181622017@stu.scu.edu.cn (M.W.); xiangliyuan@wchscu.cn (L.X.); lihonghxhx@scu.edu.cn (H.L.); 3 West China Biomedical Big Data Center, Sichuan University, Chengdu 610041, China

**Keywords:** obesity, testosterone, prostate cancer, mendelian randomization

## Abstract

**Simple Summary:**

Prostate cancer is one of the most commonly diagnosed hormone-related malignancies worldwide. Previous studies have suggested that an increased BMI is associated with a decreased risk of prostate cancer. However, it is still unclear whether testosterone plays a mediating or confounding role in the relationship between obesity-related traits and prostate cancer risk. Our study, utilizing several steps of two sample Mendelian randomization analysis, has furnished genetic evidence suggesting that serum bioavailable testosterone may mediate the effect of BMI on prostate cancer risk. This finding sheds light on a potential mechanism through which obesity could reduce the risk of prostate cancer.

**Abstract:**

Objective: This study aimed to investigate whether testosterone mediates or confounds the effect of obesity-related traits on prostate cancer (PCa) using Mendelian randomization (MR) analysis. Materials and Methods: Data of obesity-related traits (body mass index [BMI], waist-to-hip ratio [WHR], and waist-to-hip ratio adjusted for body mass index [WHRadjBMI]) were obtained from up to 806,834 people of European ancestry; data of testosterone (bioavailable testosterone [BT], total testosterone [TT], and sex hormone-binding globulin [SHBG]) were extracted from up to 194,453 participants in the UK Biobank; and the summary-level data of PCa (79,194 cases and 61,112 controls) were obtained from the PRACTICAL consortium. Result: The results supported the causal relationship between higher BMI and a reduced risk of PCa (OR = 0.91, 95% confidence interval [CI]: 0.86–0.96). Furthermore, increased BT levels were associated with an elevated risk of PCa (OR = 1.15, 95% CI: 1.06–1.24). Importantly, our analysis revealed a unidirectional causal effect—higher BMI was linked to lower BT levels (beta = −0.27, 95% CI: −0.3–−0.24), but not the other way around. This suggests that BT may mediate the effect of BMI on PCa rather than confound it. Our multivariable MR results further demonstrated that considering BT as a mediator led to the weakening of BMI’s effect on PCa risk (OR = 0.97, 95% CI: 0.90–1.05), while the impact of BT on PCa remained unchanged when accounting for BMI. Moreover, we identified a significant indirect effect of BMI on PCa risk (OR = 0.96, 95% CI: 0.94–0.98). Conclusion: Our study provided genetic evidence that serum BT can mediate the effect of BMI on the risk of PCa, indicating the possible mechanism by which obesity reduces PCa risk.

## 1. Introduction

Prostate cancer (PCa), one of the most diagnosed hormone-related malignancy worldwide, imposes significant clinical and socioeconomic burdens. In 2020 alone, there were approximately 1.4 million new cases of PCa and 375,000 PCa-related deaths, accounting for 7% of the overall cancer economic burden in the European Union [1]. Despite advancements in PCa screening [2], treatment [3], and monitoring [4] in recent years, the incidence of PCa continues to rise annually [5]. Therefore, the foundational step in developing effective primary prevention strategies for PCa is gaining a deeper understanding of its underlying causes.

Obesity, a rapidly growing public health concern, has emerged as a significant factor in cancer development [6]. In many instances, obesity-related indicators such as body mass index (BMI) have shown a clear positive correlation with cancer incidence [7,8,9]. However, when it comes to PCa, observational studies have yielded inconsistent results [10,11,12]. Recently, Mendelian randomization (MR) research has delved deeper into the genetically predicted link between BMI and PCa. While some MR studies have found no causal associations [13,14], a more comprehensive analysis, including a meta-analysis of current MR studies, has provided evidence suggesting that an increased BMI is associated with a decreased risk of PCa [15,16,17,18]. Additionally, the factors mediating this relationship and the effects of other obesity-related traits on PCa remain to be elucidated.

There is growing evidence suggesting that obesity is linked to the dysregulation of various hormonal pathways, including those involving the principal circulating androgen in males—testosterone [19]. A recent MR study has unveiled that an elevated genetically predicted BMI may lead to reduced levels of total testosterone (TT) (β = −0.25, 95% CI, −0.30 to −0.20) and bioavailable testosterone (BT) (β = −0.13; 95% CI, −0.16 to −0.09) [20]. Additionally, sex steroids such as testosterone have long been considered closely associated with PCa [21]. Considering this, we postulate that testosterone could potentially play a causal role in the link between obesity and PCa.

The primary objective of this study is to explore whether testosterone plays a mediating or confounding role in the influence of obesity-related traits on PCa through MR analysis. MR employs genetic variants strongly associated with the exposure of interest as instrumental variables (IVs) to estimate the causal effects of potentially modifiable risk factors on health outcomes [22]. Typically, traditional MR analysis can only provide an estimate of the overall causal effect, which can be further divided into the indirect effect, operating solely through mediators, and the direct effect, not reliant on mediators [23]. As an extension of MR, multivariable MR (MVMR) allows for the assessment of the direct effects of multiple related exposures on an outcome [24]. By utilizing MR and MVMR, we can concurrently evaluate causal estimates of the total, direct, and indirect effects, thereby achieving outcomes equivalent to traditional mediation analysis [23].

In this study, we utilized MR analysis to: (1) inspect whether the effect of obesity-related traits (BMI, waist-to-hip ratio [WHR], and waist-to-hip ratio adjusted for body mass index [WHRadjBMI]) on PCa remained consistent with previous MR studies; (2) estimate the causal effect of serum testosterone levels (BT, TT, and sex hormone-binding globulin [SHBG]) on PCa; (3) explore the potential bidirectional relationship between obesity-related traits and serum testosterone levels; and (4) assess whether BMI’s effect on PCa risk is mediated or confounded by serum testosterone levels.

## 2. Method

### 2.1. Study Design

This study was conducted by performing several steps of two sample MRs. The univariable MR (UVMR) was conducted in the first step to determine the causal effect of obesity-related traits on PCa, followed by the evaluation of serum testosterone levels on PCa in the second step. In subsequent steps, only those items confirmed to be causally associated with PCa were included. The direction of association between obesity-related traits and serum testosterone levels was determined by bidirectional MR in the third step, while potential mediation effects were investigated through MVMR in the fourth step. The flowgraph of each procedure is shown in Supplementary Materials Figure S1 and the STROBE-MR checklist is shown in Supplementary Materials Table S10.

### 2.2. Data Source

#### Obesity-Related Traits

The data pertaining to obesity-related traits were sourced from a genome-wide association study (GWAS) involving 806,834 individuals of European ancestry [24] (Supplementary Materials Table S1). Within this GWAS, BMI serves as a metric for overall adiposity, WHR reflects fat distribution, and WHRadjBMI characterizes fat distribution independently of overall adiposity. For the WHR measurement, waist circumference data from the UK Biobank (UKB) were divided by hip circumference. Subsequently, the WHR measure underwent regression analysis with adjustments for sex, age, age-squared at the time of evaluation, and the evaluation center. BMI was incorporated as an independent variable in this regression to compute WHRadjBMI. The BMI value was derived from standing height and weight measurements taken during the initial assessment center visit.

To establish phenotypes for the sex-specific analyses, Pulit and colleagues replicated the aforementioned procedures, tailoring the regressions separately for males and females [24]. This GWAS excluded samples without imputation data, along with samples displaying significant missingness (>5%), phenotypic-genotypic sex discrepancies, and instances of withdrawn consent.

### 2.3. Testosterone

We obtained serum testosterone level data (BT, SHBG, and TT) from a dataset encompassing up to 424,097 participants in the UK Biobank [25] (Supplementary Materials Table S1). Blood samples were collected during the initial assessment visit (2006–2010), and specific algorithms were developed to ensure the selection of aliquots that counteracted potential clustering by geographic location, collection dates, or times. This was conducted to mitigate potential biases stemming from the circadian rhythm of testosterone.

The TT level was quantified in nmol/L through a one-step competitive analysis on a Beckman Coulter Unicel Dxl 800, while the SHBG level was determined using a two-step sandwich immunoassay analysis on the same platform. Additionally, the albumin level was measured in g/L via BCG analysis on a Beckman Coulter AU5800. Calculations for the BT level were derived from testosterone, factoring in the concentration of SHBG and albumin using the Vermeulen equation in 178,782 men. Furthermore, data on SHBG levels were accessible for 180,094 men, and TT levels were obtained from 194,453 men.

The inclusion of covariates encompassed variables such as age, BMI, operation status, dilution, time of blood draw, minutes since blood draw, batch, fasting time, center, menopause, and operation status. In addition to the quality control measures undertaken by the UK Biobank, this GWAS also excluded individuals with missing values, those whose self-identified ancestry was not White European in the questionnaires, those reporting hormone-based medication use, and those with testosterone levels below the detection limit.

### 2.4. Prostate Cancer

We obtained the summary-level data on PCa from the largest-to-date GWAS with 79,194 PCa cases and 61,112 controls [26] (Supplementary Materials Table S1). The GWAS conducted genotyping through a specialized high-density genotyping array, known as the OncoArray. While the PCa status was categorized into various levels of aggressiveness, we were only able to conduct MR analyses using summary-level data of PCa due to the unavailability of individual-level data. Notably, all participants in this GWAS were males of European ancestry.

### 2.5. Statistical Analyses and Mendelian Randomization

The TwoSampleMR package (https://github.com/MRCIEU/TwoSampleMR, accessed on 23 May 2022; RRID: SCR_019010) was utilized to amalgamate and harmonize data concerning obesity-related traits, testosterone, and prostate cancer across UVMR, bidirectional MR, and MVMR analyses. The standard analysis employed was the inverse variance weighted (IVW) method with random effects. However, it is important to note that the IVW method might neglect mediated effects, potential pleiotropy, and biases stemming from causal pathways beyond the exposure. These issues can violate the instrumental variable assumptions of MR [27]. Sensitivity analyses, including MR-Egger, weighted median, and weighted mode methods, were employed to address these limitations. Furthermore, the Cochran Q statistic was employed to detect potential heterogeneity (large heterogeneity when I^2^ > 50%), and a *p*-value less than 0.05 indicated significant heterogeneity.

Horizontal pleiotropy was assessed using the intercept from the MR Egger analysis, where a *p*-value < 0.05 signaled its presence. Steiger filtering was employed to exclude SNPs that explained more outcome variation than exposure variation, mitigating the potential for false results due to pleiotropy. The MR Pleiotropy RESidual Sum and Outlier (MR-PRESSO) method was applied to identify and eliminate potential outliers in IVW regression [28].

For MVMR, an assessment of the direct effects of obesity-related traits and testosterone on PCa was conducted, accounting for the reciprocal genetic impacts between these variables. The product of coefficients method was used to evaluate the indirect effect of obesity-related traits on PCa [23].

All MR analyses were performed in R version 4.1.1 using the TwoSampleMR, MendelianRandomization, MR-PRESSO, and MVMR R packages, with two-tailed *p*-values. Causal associations were deemed suggestive when *p* < 0.05 in IVW methods, and the consistency of association direction was maintained across MR-Egger, weighted median, or weighted mode results.

### 2.6. MR Assumption and Results Interpretation

Further elaboration on the MR assumptions and the criteria employed to assess the role of mediation can be found in Supplementary Method S1. Causal effects were presented as odds ratios (OR) for binary outcomes (e.g., PCa) and as beta coefficients for continuous outcomes (such as obesity-related traits and serum testosterone levels). In instances where obesity-related traits and serum testosterone levels were treated as exposures, the unit of measurement was standardized to a scale of standard deviations (SD).

## 3. Result

### 3.1. Effect of Obesity-Related Traits on Prostate Cancer Risk

Initially, we incorporated 670 genome-wide significantly associated index SNPs (*p*-value < 5 × 10^−8^) for BMI, 316 for WHR, and 346 for WHRadjBMI. After the exclusion of variants in potential linkage disequilibrium (LD) with r^2^ ≥ 0.01 and LD distance < 10,000 kb, as well as the elimination of potential outliers via MR-PRESSO and SNPs exhibiting greater association with the outcome than the exposure using the Steiger filter, the final tally consisted of 540 SNPs for BMI, 250 for WHR, and 283 for WHRadjBMI.

**Figure 1:**
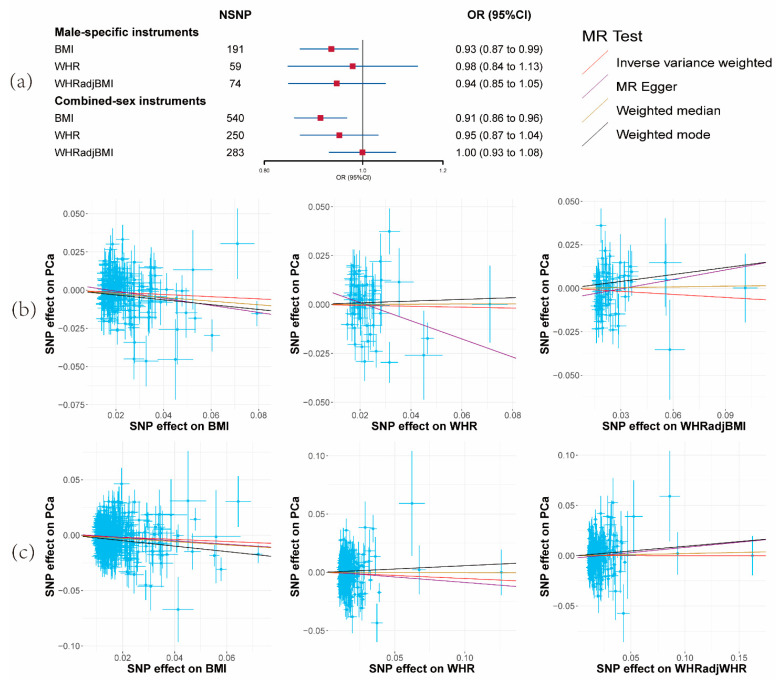
Results of the effects of obesity-related traits on prostate cancer risk. (**a**) Forest plot of the effect of obesity-related traits on prostate cancer risk with male-specific and combined-sex instruments. Effect sizes are on the odds ratio (OR) scale per 1-unit increase in exposure. (**b**) The scatter plots show the causal effect of each 1-unit change in body mass index (BMI), waist-to-hip ratio (WHR), and waist-to-hip ratio adjusted for body mass index (WHRadjBMI) on prostate cancer with male-specific instruments. (**c**) The scatter plots show the causal effect of each 1-unit change in BMI, WHR, and WHRadjBMI on prostate cancer with combined-sex instruments.

A reduced risk of PCa per unit increase in BMI was observed (OR = 0.91, 95% CI: 0.86–0.96, *p* = 0.0016) (Figure 1a and Supplementary Materials Tables S5 and S6). Nevertheless, no substantiating evidence was found for a causal effect of alterations in WHR or WHRadjBMI on PCa risk (Figure 1a and Supplementary Materials Tables S5 and S6). Correspondingly consistent outcomes emerged from the MR-Egger, weighted median, and weighted mode methods.

The subsequent utilization of sex-specific instruments for obesity-related traits resulted in findings for BMI (OR = 0.93, 95% CI: 0.87–0.99, *p* = 0.047) and the other two parameters that aligned with the main analysis, albeit with reduced statistical power (Figure 1a and Supplementary Materials Tables S5 and S6). Consequently, only BMI was retained for analysis in the third and fourth steps.

Robust instrumental variables were indicated by F-statistic values exceeding 10 across all obesity-related traits (minimum: 72; Supplementary Materials Table S2), signifying strong instruments. Notably, moderate heterogeneity was identified among BMI SNPs (I^2^ = 32.39%, Qstat = 797.29, Qpval = 2.70 × 10^−12^), WHR SNPs (I^2^ = 39.62%, Qstat = 412.36, Qpval = 3.35 × 10^−10^), and WHRadjBMI SNPs (I^2^ = 36.74%, Qstat = 445.76, Qpval = 1.69 × 10^−9^). The MR-Egger Intercept failed to furnish evidence for the presence of horizontal pleiotropy (Supplementary Materials Table S5).

### 3.2. Effect of Serum Testosterone Level on Prostate Cancer Risk

Following the removal of variants in potential linkage disequilibrium (LD) and potential outliers, a total of 62 SNPs for BT, 131 SNPs for TT, and 206 SNPs for SHBG, which displayed robust associations with the respective exposures rather than outcomes, were retained.

**Figure 2:**
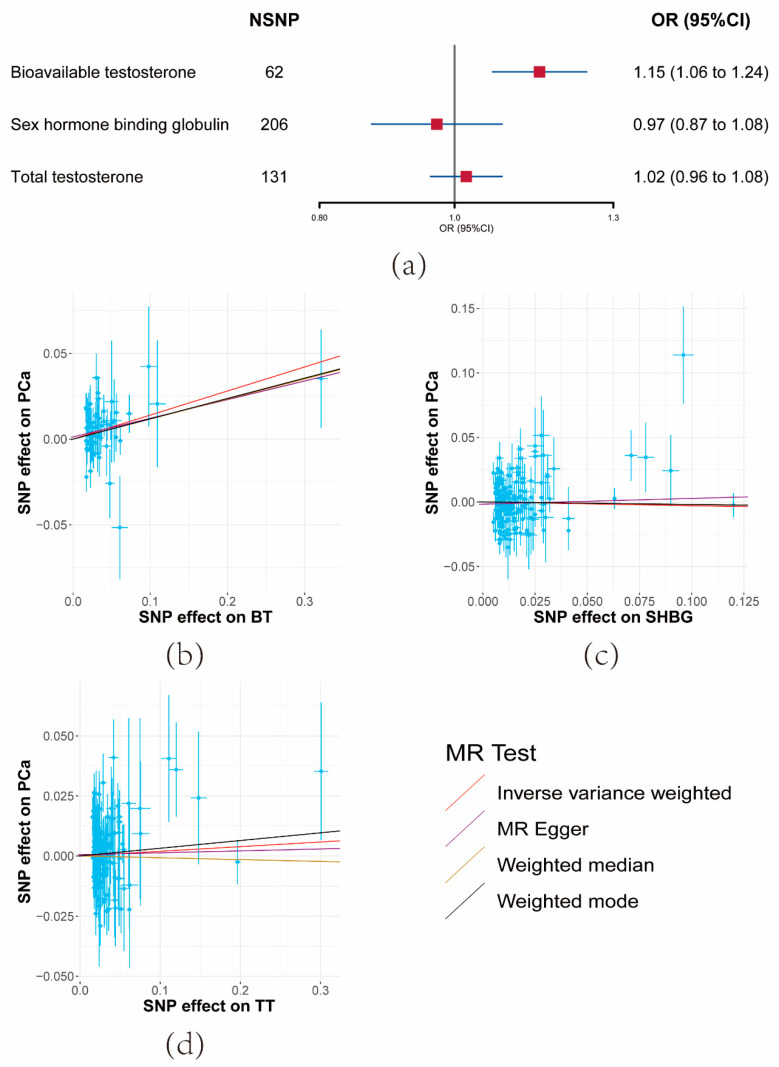
Results of the effects of serum testosterone level on prostate cancer risk. (**a**) Forest plot of the effect of serum testosterone level on prostate cancer risk. Effect sizes are on the odds ratio (OR) scale per 1-unit increase in exposure. (**b**–**d**) The scatter plots show the causal effect of each 1-unit change in (**b**) bioavailable testosterone (BT), (**c**) sex hormone-binding globulin (SHBG), and (**d**) total testosterone (TT), respectively.

The UVMR outcomes revealed that a unit increase in the serum BT level corresponded to an elevation in PCa risk (OR = 1.15, 95% CI: 1.06–1.24, *p* = 0.000404) (Figure 2a and Supplementary Materials Tables S5 and S7). Conversely, limited evidence was found to support the impact of SHBG or TT on PCa risk (Figure 2a and Supplementary Materials Tables S5 and S7). The consistent direction of association was upheld across the three sensitivity analyses. Consequently, SHBG and TT were excluded from analysis in the third and fourth steps.

F-statistics for these SNPs ranged from 37 to 41 (Supplementary Materials Table S3). Moderate heterogeneity was observed among SHBG SNPs, though not significantly among BT SNPs. The MR-Egger Intercept results indicated the absence of significant horizontal pleiotropy (Supplementary Materials Table S5).

### 3.3. Bidirectional Effects between BMI and BT

With BMI as the exposure, a total of 187 SNPs, screened out of potential linkage disequilibrium, were incorporated post-MR-PRESSO and the Steiger filter. Conversely, when BT was the exposure, 61 SNPs were included.

**Figure 3:**
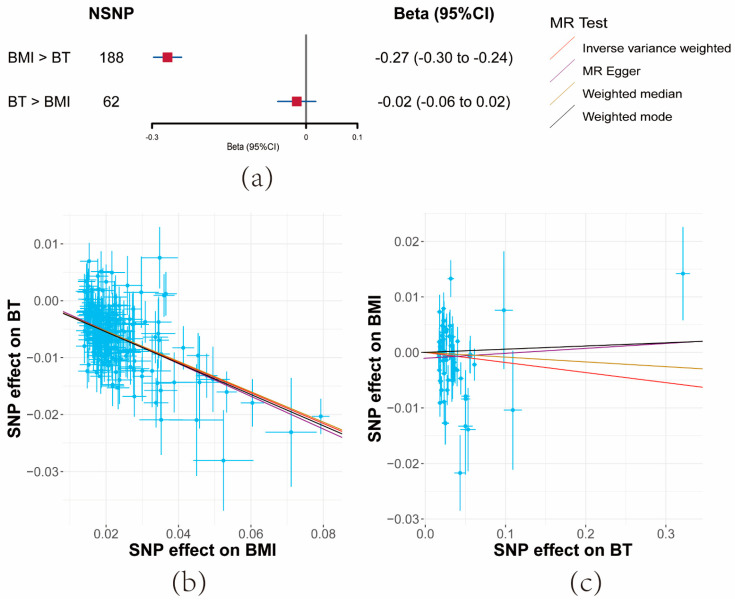
Results of bidirectional Mendelian randomization analysis between body mass index (BMI) and bioavailable testosterone (BT). (**a**) Forest plot of bidirectional effects between BMI and BT. Effect sizes are on the beta scale per 1-unit increase in exposure. (**b**,**c**) The scatter plots show the causal effect of each 1-unit change in (**b**) BMI on BT and (**c**) BT on BMI, respectively.

Both IVW and sensitivity analyses yielded consistent evidence that a unit increase in BMI significantly correlated with a decrease in serum BT levels (β = −0.27, 95% CI: −0.3–−0.24, *p* = 7.35 × 10^−84^). However, no evidence was found for the reverse causal effect of BT on BMI (β = −0.02, 95% CI: −0.06–−0.02, *p* = 0.34) (Figure 3a and Supplementary Materials Tables S5 and S8).

F-statistics for these SNPs exceeded 10 across the board (BMI: 52, BT: 36) (Supplementary Materials Table S4). While moderate heterogeneity was observed among BMI SNPs, significant heterogeneity was noted for BT. Importantly, the MR-Egger Intercept did not detect horizontal pleiotropy (Supplementary Materials Table S5).

### 3.4. Direct Effects of BMI and BT on Prostate Cancer

**Figure 4:**
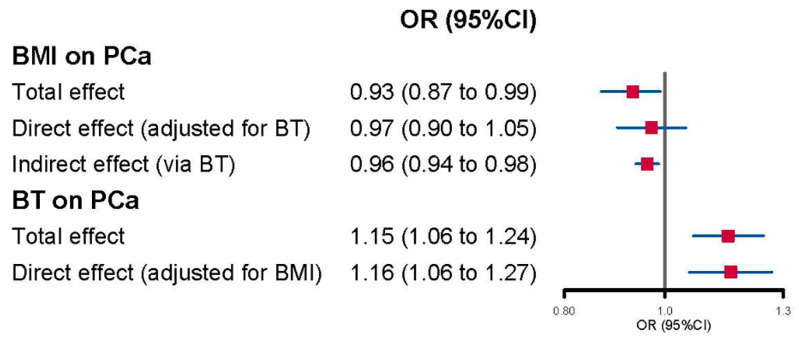
Forest plot of multivariable Mendelian randomization analysis for the effect of body mass index (BMI) and bioavailable testosterone (BT) on prostate cancer (PCa).

Upon considering BT, the direct effect estimate of BMI on PCa weakened and became statistically insignificant (OR = 0.97, 95% CI: 0.90–1.05, *p* = 0.44) when compared to the total effect (OR = 0.93, 95% CI: 0.87–0.99, *p* = 0.047) (Figure 4 and Supplementary Materials Table S9). However, the increased PCa risk associated with a unit increase in BT, initially detected in UVMR (OR = 1.15, 95% CI: 1.06–1.24, *p* = 0.000404), remained nearly unchanged when accounting for BMI (OR = 1.16, 95% CI: 1.06–1.27, *p* = 0.0021). Additionally, a significant indirect effect of BMI on PCa was also evidenced (OR = 0.96, 95% CI: 0.94–0.98) (Figure 4).

## 4. Discussion

The objective of our study was to evaluate the potential mediation role of serum testosterone levels in the relationship between obesity-related traits and PCa. Our findings have provided valuable insights into this complex interaction. To begin, our UVMR analyses revealed evidence supporting a protective effect of higher BMI, as opposed to WHR or WHRadjBMI, against PCa risk. Additionally, a higher genetically predicted BT level, but not SHBG or TT level, was associated with an increased PCa risk. Moreover, our results indicate a directional relationship where higher BMI leads to lower BT levels, while the reverse is not supported. These observations suggest that BT might indeed be situated on the causal pathway linking BMI to PCa risk. Furthermore, our MVMR analyses underscored that the direct influence of BMI on PCa risk lost significance when accounting for BT, while the effect of BT on PCa remained unchanged when considering BMI. In sum, our comprehensive findings imply that BT could potentially mediate the impact of BMI on PCa risk.

The observed effect of BMI on PCa is consistent with findings from previous MR studies [15,17]. However, our results exhibited a more conservative nature, which could be attributed to the divergent sources of instrumental variables and our stringent SNP filtering procedures. It is noteworthy that our study diverged from certain other MR investigations. For instance, some studies did not yield substantial evidence supporting the link between BMI and PCa [13], while others suggested only weak evidence indicating a decreased PCa risk with higher BMI [29]. In comparison, our study encompassed a significantly larger number of PCa cases (*n* = 79,194 vs. *n* = 1062 to 14,160) and leveraged more robustly associated BMI-related SNPs. Furthermore, unlike certain previous studies, our investigation did not furnish evidence for the influence of WHR or WHRadjBMI on PCa, a concordance with earlier research [17]. These findings prompt the consideration that the overall mass of adipose tissue, as indicated by BMI, may hold greater relevance to PCa than the specific distribution of fat.

Our UVMR findings provided support for the assertion that a higher genetically predicted BT level represented a risk factor for PCa, while evidence for the effect of TT and SHBG was limited. These outcomes align with the perspective that BT exhibits a more robust association with specific androgen-dependent outcomes compared to TT [30]. In observational studies, the relationship between testosterone levels and PCa remains contentious, often due to limitations in sample size, abbreviated follow-up periods, and inconsistent respondent age [31,32]. By overcoming unmeasured confounding prevalent in observational research, our findings, along with prior MR analyses, furnish more resilient evidence that lifelong exposure to elevated genetically predicted BT levels (but not TT or SHBG) may indeed lead to an increased PCa risk [25,33].

The outcomes from our bidirectional MR analysis, demonstrating that BMI could lead to a decrease in BT levels, echoed the findings of both real-world observational studies [34] and previous MR analyses [20]. This reaffirms the commonly observed association between increased adiposity and impaired testosterone levels. Turning to the influence of BT on BMI, the results from observational studies have been varied due to factors like study population heterogeneity (e.g., hypogonadal men or transgender men) and differences in follow-up duration [35,36]. Conversely, the outcomes of a randomized controlled trial (RCT) aligned with our perspective, revealing no significant changes in BMI for patients using testosterone over a 3-month period [37]. Our findings also harmonized with those of another bidirectional MR study, which offered limited evidence to support the notion that long-term changes in testosterone could affect BMI in the general population [38].

The unidirectional impact of BMI on BT implies that BT is more likely to act as a mediator in the relationship between BMI and PCa. Meanwhile, the limited evidence regarding the effect of BT on BMI suggests that BT might not be a confounding factor in the BMI-PCa relationship. The findings from the fourth step of analysis corroborate this perspective: (1) the direct effect of BMI on PCa weakened to insignificance when BT was taken into account, while the effect of BT on PCa remained unchanged when BMI was considered; (2) the indirect effect of BMI (operating solely through the hypothesized mediator BT) on PCa was significant. In reality, while not conclusively demonstrated in observational studies, the mediating role of testosterone in the connection between obesity and PCa risk has been suggested. For instance, Giovannucci and colleagues discovered that men with higher BMIs (≥30 kg/m^2^) had a notably reduced PCa risk [10], positing that the mechanism might involve the lower circulating testosterone levels often observed in obesity. Another study supported the idea that changes in hormone levels related to obesity could mediate the decline in PSA levels, although it excluded men with a history of PCa [39]. Our study complements these existing findings and goes a step further, highlighting that BT, distinct from TT or SHBG, might mediate the influence of BMI (but not WHR or WHRadjBMI) on PCa.

Our study possesses several notable strengths. Firstly, we employed a comprehensive approach encompassing UVMR, bidirectional MR, and MVMR methodologies, allowing us to assess mediation effects while concurrently distinguishing potential confounding roles. Utilizing the MR framework for mediation analysis addresses the limitations inherent in conventional mediation analysis, where accurate model specification involving the assignment of variables to mediators or confounders is pivotal for precise mediation assessment [23]. Secondly, our study integrated various sensitivity analyses and SNP-filtering procedures throughout the analytical process, thereby mitigating potential pleiotropy bias and ensuring the robustness of SNP selection. Thirdly, our utilization of genetic variant data from the largest-to-date available GWAS contributes to the clinical relevance and significance of our findings. However, our study also bears certain limitations. Firstly, as we relied on genetic variants to predict PCa risk, our results might be more applicable to PCa cases with inherent susceptibility. Secondly, there is partial overlap between the samples of the GWAS for obesity-related traits and testosterone in the UK Biobank dataset, which could introduce a bias known as “the winner’s curse”. Lastly, the genetic variant data solely originate from individuals of European ancestry, warranting further investigation involving diverse ancestries to establish more generalized conclusions.

## 5. Conclusions

Our study contributes genetic evidence supporting the role of serum BT as a mediator in the causal relationship between BMI and PCa risk. Furthermore, we provide additional insights into the effects of both BMI and BT on PCa risk. These findings shed light on a potential mechanism through which obesity might influence PCa risk. Future larger-scale interventional studies are necessary to validate and confirm the implications of our results.

## Data Availability

The data used in this study were publicly available and can be accessed via the following links: (1) The genetic instruments come from the published GWAS study as described in the method section. (2) Analyses are performed using the TwoSampleMR package, and the code is available on the website (https://mrcieu.github.io/TwoSampleMR/reference/index.html, accessed on 23 May 2022). The RRID for the TwoSampleMR package is SCR_019010.

## Supplementary Materials

The following supporting information can be downloaded at: https://www.mdpi.com/article/10.3390/cancers15194884/s1. Supplementary Method S1: MR assumption and results interpretation [40,41]; Supplementary Table S1: The characteristics of GWAS summary statistics used in main analyses; Supplementary Table S2: Proportion of variance explained and F statistics for obesity-related traits on prostate cancer; Supplementary Table S3: Proportion of variance explained and F statistics for serum testosterone on prostate cancer; Supplementary Table S4: Proportion of variance explained and F statistics for bidirectional MR between obesity-related traits and serum testosterone; Supplementary Table S5: Results of the main analysis for univariable MR and bidirectional MR; Supplementary Table S6: Results for obesity-related traits effect on prostate cancer; Supplementary Table S7: Results for serum testosterone effect on prostate cancer; Supplementary Table S8: Results for bidirectional MR between BMI and BT; Supplementary Table S9: Results for MVMR; Supplementary Table S10: STROBE-MR checklist of recommended items to address in reports of Mendelian randomization studies [42]; Supplementary Figure S1: Overview of the study design.
