# Supplementary material for: Do Obesity-Related Traits Affect Prostate Cancer Risk through Serum Testosterone? A Mendelian Randomization Study
Source: Cancers (Basel). 2023 Oct 8;15(19):4884. doi: 10.3390/cancers15194884 (PMC10571835; doi:10.3390/cancers15194884)
Supplement: Supplementary file 1 [file cancers-15-04884-s001.zip › cancers-2595110-supplementary.pdf]

Supplementary Method S1:

MR assumption and results interpretation

The MR analysis relies on three important assumptions to get robust results: 1) relevance: the IVs must be robustly related to the exposure, (2) independence: the IVs must not be related to confounders, and (3) exclusion restriction: IVs must only influence the outcome via the exposure. We only selected genome-wide significant associated (P-value $< 5 \times 10^{-8}$) index SNPs to meet assumption 1. Besides, the calculated F-statistics for obesity-related traits were all greater than 10 (supplementary table 2-4), which minimized the possibility of weak instrumental variables [1]. Additionally, we excluded one of each pair of genetic variants that are in linkage disequilibrium (LD) ($r^2 < 0.01$, LD distance > 10,000kb), and conduct additional sensitivity analyses (MR-Egger, weighted median, and weighted mode methods), Steiger filtering and MR-PRESSO for reduction of pleiotropic effects (assumptions 2 and 3) [2]. Moreover, we estimated the heterogeneity using Cochran's Q statistic to further test assumptions 2 and 3, since the existence of heterogeneity may result from the pleiotropy of SNPs.

If the results met the following criteria, we would consider the existence of a potential mediating role: 1) a causal effect of a specific obesity-related anthropometric item on PCa was demonstrated in the first step; 2) a causal effect of specific serum testosterone on PCa was demonstrated in the second step; 3) a unidirectional causal effect of a specific obesity-related anthropometric item on the specific serum testosterone was indicated in the third step; 4) the causal effect of a specific obesity-related anthropometric item on PCa were weakened when adjusted for specific serum testosterone, while the causal effect of specific serum testosterone on PCa remain consistent when adjusted for a specific obesity-related anthropometric in the fourth step. 5) an indirect causal effect, but not a direct causal effect of a specific obesity-related anthropometric item on PCa, and an indirect causal effect of specific serum testosterone was confirmed in the fourth step.

[1] Burgess S, Thompson S G. Avoiding bias from weak instruments in Mendelian randomization studies [J]. Int J Epidemiol, 2011, 40(3): 755-764.
[2] Andersen M L, Alvarenga T F, Mazaro-Costa R, et al. The association of testosterone, sleep, and sexual function in men and women [J]. Brain Res, 2011, 1416.

Supplementary Table S1: The characteristics of GWAS summary statistics used in main analyses.

GWAS, genome-wide association study; SNP, single nucleotide polymorphism; SD, standard deviation; BMI, Body Mass Index; WHR, waist-to-hip ratio; WHRadjBMI, waist-to-hip ratio adjusted for body mass index; GIANT; Genetic Investigation of ANthropometric Traits; UKB, UK biobank; PRACTICAL, Prostate Cancer Association Group to Investigate Cancer-Associated Alterations in the Genome; BT, Bioavailable testosterone; TT, total testosterone; SHBG, sex hormone-binding globulin; SD, standard deviation

Items	Subitems	Population	Trait or case definition	Adjusted covariates	PMID	Units	Sample size in UK Biobank
Exposure							
Obesity-related traits	BMI	European: 806,834 individuals (including 374,756 males) of GIANT consortium and UKB	BMI = kg/m^2 where kg is a person's weight in kilograms and m^2 is their standing height in metres squared.	sex, age, and age-squared at the time of evaluation, and the evaluation center	30239722	SD	484,680 individuals including 221,863 males
	WHR	European: 697,734 individuals (including 316,772 males) of GIANT consortium and UKB	the waist circumference data from UKB was divided by hip circumference	sex, age, and age-squared at the time of evaluation, and the evaluation center	30239722	SD	485,486 individuals including 222,338 males
	WHRadjBMI	European: 694,649 individuals (including 315,284 males) of GIANT consortium and UKB	Regress the WHR measure on BMI, sex, age, and age-squared at the time of evaluation, and the evaluation center.	BMI, sex, age, and age-squared at the time of evaluation, and the evaluation center	30239722	SD	484,563 individuals including 221,804 males
Mediator							
Serum testosterone level	Bioavailable testosterone	European: 178,782 males	From blood sample collected at the initial visit. Testosterone was measured in nmol/L by one-step competitive analysis on a Beckman Coulter Unicel	Fasting time, age, centre, chip/release of genetic data	32042192	SD	178,782

				Dxl 800. SHBG was measured in nmol/L by two-step sandwich immunoassay analysis on a Beckman Coulter Unicel Dxl 800. Albumin measured in g/L by BCG analysis on a Beckman Coulter AU5800.				
	SHBG	European: males	180,094	From blood sample collected at the initial visit. Measured by two-step sandwich immunoassay analysis on a Beckman Coulter Unicel Dxl 800	Age, BMI, batch, dilution	32042192	SD	180,094
	Total testosterone	European: males	194,453	From blood sample collected at the initial visit. Measured in nmol/L by one-step competitive analysis on a Beckman Coulter Unicel Dxl 800.	Fasting time, age, centre, chip/release of genetic data	32042192	SD	194,453
Outcome								
Prostate cancer		European: PRACTICAL consortium including 79,194 PCa cases and 61,112 controls		low aggressive (tumor stage <=T1 and Gleason score [GS] <=6 and PSA<10), intermediate aggressive (T2 or GS7 or PSA 10-20), high aggressive (T3/T4 or N1 or M1 or GS>=8 or PSA>20) and advanced (metastatic disease or GS>=8 or PSA>100 or PCa death)	ancestry, country, principal components, and so forth	29892016	/	0

Supplementary Table S2: Proportion of variance explained and F statistics for obesity-related traits on prostate cancer.

BMI, Body Mass Index; WHR, waist-to-hip ratio; WHRadjBMI, waist-to-hip ratio adjusted for body mass index; SNP, single nucleotide polymorphism

Variables	The sample size of exposure	The sample size of the outcome	SNPs	The proportion of variance explained	F statistics
Male-specific instrument					
BMI	374,765	79,194	191	2.57%	52
WHR	316,772	79,194	59	0.81%	44
WHRadjBMI	315,284	79,194	74	1.04%	48
Combined-sex instrument					
BMI	806,834	79,194	540	5.01%	79
WHR	697,734	79,194	250	2.51%	72
WHRadjBMI	694,649	79,194	283	2.98%	75

The proportion of variance in the exposure explained by the genetic variants was calculated using the TwoSampleMR R functions get_r_from_pn ().

The F statistics related to the proportion of variance in the exposure explained by the genetic variants(R2), sample size(N), and the number of instruments(K) were calculated by the formula F = ((N-K-1)/K) (R2/(1-R2)).

Supplementary Table S3: Proportion of variance explained and F statistics for serum testosterone on prostate cancer.

SNP, single nucleotide polymorphism

Variables	The sample size of exposure	The sample size of the outcome	SNPs	The proportion of variance explained	F statistics
bioavailable testosterone	178,782	79,194	62	1.29%	38
sex hormone-binding globulin	180,094	79,194	206	4.51%	41
total testosterone	194,453	79,194	131	2.44%	37

the proportion of variance in the exposure explained by the genetic variants was calculated using the TwoSampleMR R functions get_r_from_pn ().

The F statistics related to the proportion of variance in the exposure explained by the genetic variants(R2), sample size(N), and the number of instruments(K) were calculated by the formula F = ((N-K-1)/K) (R2/(1-R2)).

Supplementary Table S4: Proportion of variance explained and F statistics for bidirectional MR between obesity-related traits and serum testosterone.
SNP, single nucleotide polymorphism

Variables	The sample size of exposure	The sample size of the outcome	SNPs	The proportion of variance explained	F statistics
BMI	374,765	178,782	188	2.52%	52
bioavailable testosterone	178,782	374,765	62	1.24%	36

the proportion of variance in the exposure explained by the genetic variants was calculated using the TwoSampleMR R functions get_r_from_pn ().

The F statistics related to the proportion of variance in the exposure explained by the genetic variants(R2), sample size(N), and the number of instruments(K) were calculated by the formula F = ((N-K-1)/K) (R2/(1-R2)).

Supplementary Table S5: Results of the main analysis for univariable MR and bidirectional MR.

BMI, Body Mass Index; WHR, waist-to-hip ratio; WHRadjBMI, waist-to-hip ratio adjusted for body mass index; PCa, prostate cancer; SNP, single nucleotide polymorphism; IVW, inverse variance weighted; MR-PRESSO, MR Pleiotropy RESidual Sum and Outlier; UVMR, univariable MR; BT, Bioavailable testosterone; TT, total testosterone; SHBG, sex hormone-binding globulin; SD, standard deviation

Exposures	Outcome	SNPs*	IVW OR beta p-value	IVW p-value	Q	Q_df	IVW SNP heterogeneity p-value	IVW SNP heterogeneity I^2	SNPs**	IVW after MR-PRESSO OR/ Beta (95% CI)	IVW after MR-PRESSO p-value***	Q	Q_df	IVW after MR-PRESSO SNP heterogeneity p-value	IVW after MR-PRESSO SNP heterogeneity I^2	MR-Egger Intercept p-value
UVMR: Male-specific instrument																
BMI	PCa	193	0.94 (0.88-1.01)	0.11	310.673	192	1.25E-07	38.20%	191	0.93(0.87-0.99)	0.047	288.5933741	190	5.13E-06	34.16%	0.071908
WHR	PCa	64	1.05 (0.88-1.25)	0.59	196.0003	63	1.46E-15	67.86%	59	0.98(0.84-1.13)	0.76	113.3594229	58	1.90E-05	48.84%	0.080695
WHRadjBMI	PCa	79	0.99(0.87-1.14)	0.96	194.029	78	7.24E-12	59.80%	74	0.94(0.84-1.05)	0.29	106.0865952	73	0.006916248	31.19%	0.155668
UVMR: Combined-sex instrument																
BMI	PCa	545	0.91(0.86-0.97)	0.0038	863.7619	544	6.14E-17	37.02%	540	0.91(0.86-0.96)	0.0016	797.2781599	539	2.70E-12	32.39%	0.440606
WHR	PCa	253	0..97(0.88-1.06)	0.47	450.5407	252	2.12E-13	44.07%	250	0.95(0.87-1.04)	0.24	412.3618187	249	3.35E-10	39.62%	0.726876
WHRadjBMI	PCa	287	1.02(0.94-1.11)	0.58	538.8113	286	1.02E-17	46.92%	283	1.00(0.93-1.08)	0.99	445.7558881	282	1.69E-09	36.74%	0.22656
UVMR																
BT	PCa	65	1.20 (1.09-1.33)	0.00037	134.1637	64	6.86E-07	52.30%	62	1.15 (1.06-1.24)	0.000404	74.32260959	61	0.117521678	17.93%	0.635638
SHBG	PCa	217	0.89(0.78-1.03)	0.11	724.0862	216	4.14E-56	70.17%	206	0.97 (0.87-1.08)	0.594901797	378.4357325	205	1.93E-12	45.83%	0.218332
TT	PCa	141	0.98(0.91-1.06)	0.69	407.3853	140	6.33E-28	65.63%	131	1.02 (0.96-1.08)	0.520159003	205.7216959	130	2.60E-05	36.81%	0.781029
Bidirectional MR																
BMI	BT	198	-0.24(-0.28--0.21)	2.12E-45	506.6745	197	3.71E-29	61.12%	188	-0.27(-0.3 - -0.24)	7.35E-84	298.2227699	187	4.19E-07	37.30%	0.622068
BT	BMI	69	-0.019 (-0.071 -0.033)	0.48	401.4825	68	8.81E-49	83.06%	62	-0.02 (-0.06 - 0.02)	0.335811504	155.1648993	61	3.75E-10	60.69%	0.372557

Supplementary Table S6: Results for obesity-related traits effect on prostate cancer.

BMI, Body Mass Index; WHR, waist-to-hip ratio; WHRadjBMI, waist-to-hip ratio adjusted for body mass index; PCa, prostate cancer; SNP, single nucleotide polymorphism; IVW, inverse variance weighted; MR-PRESSO, MR Pleiotropy RESidual Sum and Outlier; UVMR, univariable MR; BT, Bioavailable testosterone; TT, total testosterone; SHBG, sex hormone-binding globulin; SD, standard deviation; OR, odds ratio; se, standard error; ci, confidence interval;

exposure	outcome	method	nsnp	b	se	pval	lo_ci	up_ci	or	or_lci95	or_uci95
**Male-specific instrument——before MR-PRESSO**											
BMI	Prostate Cancer	Inverse variance weighted	193	-0.05931	0.037004	0.108965	-0.13184	0.013215	0.942413	0.876482	1.013303
		MR Egger	193	-0.2293	0.099053	0.021682	-0.42344	-0.03515	0.795092	0.654789	0.965457
		Weighted median	193	-0.11766	0.051358	0.021968	-0.21832	-0.017	0.889	0.803868	0.983148
		Weighted mode	193	-0.15862	0.083487	0.058942	-0.32225	0.005016	0.853322	0.724515	1.005028
**Male-specific instrument——after MR-PRESSO**											
BMI	Prostate Cancer	Inverse variance weighted	191	-0.07122	0.035988	0.04782	-0.14175	-0.00068	0.931259	0.867835	0.999318
		MR Egger	191	-0.23245	0.096003	0.016409	-0.42062	-0.04429	0.792585	0.656639	0.956677
		Weighted median	191	-0.11981	0.050138	0.016868	-0.21808	-0.02154	0.88709	0.804061	0.978692
		Weighted mode	191	-0.15805	0.091004	0.084055	-0.33642	0.020318	0.853808	0.714326	1.020526
**Male-specific instrument——before MR-PRESSO**											
WHR	Prostate Cancer	Inverse variance weighted	64	0.047859	0.0902	0.595704	-0.12893	0.224651	1.049023	0.879033	1.251886
		MR Egger	64	-0.50504	0.322615	0.122563	-1.13737	0.127282	0.603479	0.320662	1.135737
		Weighted median	64	0.016299	0.085368	0.848585	-0.15102	0.183621	1.016432	0.859828	1.20156
		Weighted mode	64	0.030701	0.163028	0.851233	-0.28883	0.350236	1.031177	0.749137	1.419402
**Male-specific instrument——after MR-PRESSO**											
WHR	Prostate Cancer	Inverse variance weighted	59	-0.02242	0.075052	0.765194	-0.16952	0.124687	0.977834	0.844072	1.132793
		MR Egger	59	-0.46451	0.259303	0.078542	-0.97275	0.043723	0.628442	0.378044	1.044693
		Weighted median	59	0.0046	0.090986	0.959674	-0.17373	0.182933	1.004611	0.840522	1.200734
		Weighted mode	59	0.043737	0.189541	0.81832	-0.32776	0.415238	1.044707	0.720533	1.514731
**Male-specific instrument——before MR-PRESSO**											
WHRadjBMI	Prostate Cancer	Inverse variance weighted	79	-0.00359	0.070594	0.959405	-0.14196	0.134772	0.996413	0.867658	1.144275
		MR Egger	79	0.414738	0.218947	0.061949	-0.0144	0.843874	1.513974	0.985705	2.325358
		Weighted median	79	0.02577	0.070527	0.714822	-0.11246	0.164003	1.026105	0.89363	1.178218
		Weighted mode	79	0.119726	0.118363	0.314897	-0.11227	0.351718	1.127188	0.893807	1.421508
**Male-specific instrument——after MR-PRESSO**											
WHRadjBMI	Prostate Cancer	Inverse variance weighted	74	-0.05841	0.056118	0.297954	-0.1684	0.051582	0.943264	0.845015	1.052936

		MR Egger	74	0.180788	0.175773	0.307143	-0.16373	0.525304	1.198161	0.848974	1.690972
		Weighted median	74	0.013498	0.068747	0.844345	-0.12125	0.148243	1.013589	0.885815	1.159794
		Weighted mode	74	0.132231	0.119585	0.272465	-0.10216	0.366618	1.141372	0.902889	1.442846
**Combined-sex instrument——before MR-PRESSO**											
BMI	Prostate Cancer	Inverse variance weighted	545	-0.09013	0.031168	0.003831	-0.15122	-0.02904	0.913812	0.859658	0.971377
		MR Egger	545	-0.14278	0.08327	0.086985	-0.30599	0.020433	0.866947	0.736396	1.020643
		Weighted median	545	-0.14227	0.045886	0.001932	-0.23221	-0.05233	0.867388	0.792783	0.949013
		Weighted mode	545	-0.24788	0.088228	0.005139	-0.42081	-0.07496	0.780451	0.656515	0.927784
**Combined-sex instrument——after MR-PRESSO**											
BMI	Prostate Cancer	Inverse variance weighted	540	-0.09543	0.030304	0.001639	-0.15482	-0.03603	0.908986	0.856568	0.964613
		MR Egger	540	-0.15317	0.080736	0.058335	-0.31142	0.005069	0.857981	0.732409	1.005082
		Weighted median	540	-0.14672	0.046594	0.001638	-0.23805	-0.0554	0.863532	0.788164	0.946107
		Weighted mode	540	-0.24734	0.081488	0.002519	-0.40706	-0.08763	0.780874	0.665605	0.916104
**Combined-sex instrument——before MR-PRESSO**											
WHR	Prostate Cancer	Inverse variance weighted	253	-0.03395	0.046639	0.466662	-0.12536	0.057463	0.96662	0.882177	1.059146
		MR Egger	253	-0.13373	0.130314	0.305782	-0.38914	0.121686	0.874827	0.677637	1.129399
		Weighted median	253	-0.00116	0.063721	0.985453	-0.12605	0.123731	0.998839	0.881566	1.131712
		Weighted mode	253	0.051024	0.1291	0.693007	-0.20201	0.30406	1.052348	0.817086	1.35535
**Combined-sex instrument——after MR-PRESSO**											
WHR	Prostate Cancer	Inverse variance weighted	250	-0.05304	0.045064	0.239155	-0.14137	0.035281	0.948337	0.868168	1.03591
		MR Egger	250	-0.09413	0.125879	0.455279	-0.34086	0.152588	0.910161	0.711161	1.164845
		Weighted median	250	-0.00225	0.065093	0.972388	-0.12984	0.125329	0.997749	0.87824	1.133521
		Weighted mode	250	0.056947	0.126666	0.653401	-0.19132	0.305213	1.0586	0.82587	1.356914
**Combined-sex instrument——before MR-PRESSO**											
WHRadjBMI	Prostate Cancer	Inverse variance weighted	287	0.022993	0.041654	0.58094	-0.05865	0.104635	1.02326	0.943039	1.110305
		MR Egger	287	0.132865	0.102163	0.194475	-0.06737	0.333104	1.142095	0.934845	1.395292
		Weighted median	287	0.021965	0.058658	0.708058	-0.093	0.136934	1.022208	0.91119	1.146753
		Weighted mode	287	0.093761	0.082805	0.258453	-0.06854	0.256058	1.098297	0.933759	1.291828
**Combined-sex instrument——after MR-PRESSO**											
WHRadjBMI	Prostate Cancer	Inverse variance weighted	283	-0.00028	0.038449	0.994116	-0.07564	0.075077	0.999716	0.927146	1.077967
		MR Egger	283	0.10339	0.093775	0.271174	-0.08041	0.28719	1.108924	0.922739	1.332677
		Weighted median	283	0.02127	0.057304	0.710505	-0.09105	0.133587	1.021498	0.912975	1.14292
		Weighted mode	283	0.093309	0.08259	0.259527	-0.06857	0.255184	1.0978	0.933731	1.290699

Supplementary Table S7: results for serum testosterone effect on prostate cancer.

SNP, single nucleotide polymorphism; IVW, inverse variance weighted; MR-PRESSO, MR Pleiotropy RESidual Sum and Outlier; UVMR, univariable MR; BT, Bioavailable testosterone; TT, total testosterone; SHBG, sex hormone-binding globulin; SD, standard deviation; OR, odds ratio; se, standard error; ci, confidence interval;

exposure	outcome	method	nsnp	b	se	pval	lo_ci	up_ci	or	or_lci95	or_uci95
**before MR-PRESSO**											
BT	Prostate Cancer	Inverse variance weighted	65	0.18312986	0.05144688	0.00037143	0.08229399	0.28396574	1.20097036	1.08577497	1.32838742
		MR Egger	65	0.08230088	0.10032903	0.41513256	-0.114344	0.27894577	1.08578245	0.89195106	1.32173567
		Weighted median	65	0.11853061	0.05766894	0.03984395	0.00549949	0.23156174	1.12584134	1.00551464	1.26056716
		Weighted mode	65	0.11811317	0.05727051	0.0432363	0.00586298	0.23036336	1.12537146	1.0058802	1.25905742
**after MR-PRESSO**											
BT	Prostate Cancer	Inverse variance weighted	62	0.14042161	0.0396999	0.00040458	0.0626098	0.21823343	1.15075887	1.06461135	1.24387739
		MR Egger	62	0.10880701	0.07748061	0.16538127	-0.043055	0.26066902	1.11494716	0.95785871	1.29779805
		Weighted median	62	0.11710095	0.05908073	0.04747367	0.00130272	0.23289919	1.12423292	1.00130357	1.26225423
		Weighted mode	62	0.11860694	0.06006062	0.05282333	0.00088813	0.23632575	1.12592727	1.00088852	1.26658684
**before MR-PRESSO**											
SHBG	Prostate Cancer	Inverse variance weighted	217	-0.1130695	0.07117861	0.11216612	-0.2525796	0.02644058	0.89308859	0.7767944	1.02679324
		MR Egger	217	-0.0290163	0.10502611	0.78260094	-0.2348674	0.17683491	0.97140066	0.79067565	1.19343406
		Weighted median	217	-0.020676	0.07331094	0.7779191	-0.1643655	0.12301342	0.97953627	0.84843191	1.13089959
		Weighted mode	217	-0.024377	0.06458585	0.70622096	-0.1509652	0.1022113	0.97591776	0.85987761	1.10761749
**after MR-PRESSO**											
SHBG	Prostate Cancer	Inverse variance weighted	206	-0.029259	0.05502433	0.5949018	-0.1371067	0.07858871	0.97116492	0.87187723	1.08175931
		MR Egger	206	0.04449646	0.08116527	0.58413996	-0.1145875	0.20358038	1.04550127	0.89173394	1.22578368
		Weighted median	206	-0.0205699	0.07383961	0.7805705	-0.1652956	0.12415572	0.9796402	0.84764314	1.13219216
		Weighted mode	206	-0.0194254	0.06419688	0.762508	-0.1452513	0.10640045	0.98076203	0.86480494	1.11226719
**before MR-PRESSO**											
TT	Prostate Cancer	Inverse variance weighted	141	-0.0153019	0.03932854	0.69721861	-0.0923858	0.06178207	0.98481462	0.91175334	1.0637305
		MR Egger	141	-0.0321726	0.0652947	0.62298142	-0.1601502	0.09580502	0.96833943	0.85201579	1.10054445
		Weighted median	141	-0.0086685	0.04272722	0.83922807	-0.0924139	0.0750768	0.99136892	0.91172772	1.07796694
		Weighted mode	141	0.02659905	0.04206222	0.52817405	-0.0558429	0.109041	1.02695596	0.94568768	1.11520808
**after MR-PRESSO**											
TT	Prostate Cancer	Inverse variance weighted	131	0.01937003	0.03011976	0.520159	-0.0396647	0.07840476	1.01955885	0.96111164	1.08156035
		MR Egger	131	0.00801573	0.05074569	0.87473626	-0.0914458	0.10747729	1.00804794	0.91261075	1.11346557

Weighted median	131	-0.0075806	0.04307262	0.86029769	-0.0920029	0.07684176	0.99244808	0.91210249	1.07987118
Weighted mode	131	0.03213156	0.04244494	0.45040926	-0.0510605	0.11532364	1.03265335	0.95022116	1.12223658

Supplementary Table S8: results for bidirectional MR between BMI and BT.

exposure	outcome	method	nsnp	b	se	pval	lo_ci	up_ci	or	or_lci95	or_uci95
**before MR-PRESSO**											
BMI	BT	Inverse variance weighted	198	-0.2440218	0.01725609	2.12E-45	-0.2778438	-0.2101999	0.78347052	0.75741514	0.81042222
		MR Egger	198	-0.2491881	0.04715163	3.34E-07	-0.3416053	-0.1567709	0.77943333	0.71062863	0.85489987
		Weighted median	198	-0.2655171	0.02169006	1.87E-34	-0.3080296	-0.2230046	0.76680934	0.73489356	0.80011119
		Weighted mode	198	-0.2725041	0.02861992	6.39E-18	-0.3285991	-0.216409	0.76147032	0.71993156	0.8054058
**after MR-PRESSO**											
BMI	BT	Inverse variance weighted	188	-0.2698282	0.01390685	7.35E-84	-0.2970856	-0.2425707	0.76351068	0.74298042	0.78460825
		MR Egger	188	-0.2872297	0.03789887	1.60E-12	-0.3615115	-0.212948	0.75033932	0.69662256	0.8081982
		Weighted median	188	-0.2666786	0.01993007	7.84E-41	-0.3057416	-0.2276157	0.76591916	0.73657693	0.79643027
		Weighted mode	188	-0.2747692	0.0269502	1.09E-19	-0.3275916	-0.2219468	0.75974742	0.72065725	0.80095793
**before MR-PRESSO**											
BT	BMI	Inverse variance weighted	69	-0.0187263	0.02664502	0.4821752	-0.0709506	0.03349789	0.98144791	0.93150793	1.03406527
		MR Egger	69	0.0416628	0.05099848	0.41685703	-0.0582942	0.14161981	1.04254287	0.94337235	1.15213853
		Weighted median	69	-0.003611	0.01935627	0.85201065	-0.0415493	0.03432731	0.99639553	0.95930207	1.03492329
		Weighted mode	69	0.00925942	0.02065276	0.65533441	-0.03122	0.04973882	1.00930242	0.96926233	1.05099656
**after MR-PRESSO**											
BMI	BT	Inverse variance weighted	62	-0.0181813	0.01889013	0.3358115	-0.0552059	0.0188434	0.98198302	0.94629027	1.01902205
		MR Egger	62	0.0089063	0.0355952	0.80327928	-0.0608603	0.07867289	1.00894607	0.94095468	1.08185039
		Weighted median	62	-0.0085762	0.01993873	0.66710383	-0.0476561	0.03050374	0.9914605	0.95346165	1.03097374
		Weighted mode	62	0.00576069	0.01978838	0.77195103	-0.0330245	0.04454592	1.00577732	0.96751482	1.04555299

Supplementary Table S9: Results for MVMR.

BMI, Body Mass Index; SNP, single nucleotide polymorphism; IVW, inverse variance weighted; BT, Bioavailable testosterone; OR, odds ratio; se, standard error; ci, confidence interval;

exposure	outcome	method	or	or_lci95	or_uci95	P value
BMI (adjusted for BT)	PCa	IVW	0.97040967	0.89950517	1.0469033	0.43859528
BT (adjusted for BMI)	Pca	IVW	1.15669063	1.05524373	1.26789023	0.00212183

Supplementary Table S10: STROBE-MR checklist of recommended items to address in reports of Mendelian randomization studies.[i]

Item No.	Section		Checklist item	Page No.	Relevant text from manuscript
1	**TITLE and ABSTRACT**		Indicate Mendelian randomization (MR) as the study's design in the title and/or the abstract if that is a main purpose of the study	**1-2**	**Line 1-63**
	**INTRODUCTION**				
2	**Background**		Explain the scientific background and rationale for the reported study. What is the exposure? Is a potential causal relationship between exposure and outcome plausible? Justify why MR is a helpful method to address the study question	**3**	**Line 67-102**
3	**Objectives**		State specific objectives clearly, including pre-specified causal hypotheses (if any). State that MR is a method that, under specific assumptions, intends to estimate causal effects	**3**	**Line 104-109**
	**METHODS**				
4	**Study design and data sources**		Present key elements of the study design early in the article. Consider including a table listing sources of data for all phases of the study. For each data source contributing to the analysis, describe the following:	**4-6**	**Line 113-228**
		a)	Setting: Describe the study design and the underlying population, if possible. Describe the setting, locations, and relevant dates, including periods of recruitment, exposure, follow-up, and data collection, when available.	**4**	**Line 115-124**
		b)	Participants: Give the eligibility criteria, and the sources and methods of selection of participants. Report the sample size, and whether any power or sample size calculations were carried out prior to the main analysis	**4-5**	**Line 127-170**
		c)	Describe measurement, quality control and selection of genetic variants	**6**	**Line 203-214**
		d)	For each exposure, outcome, and other relevant variables, describe methods of assessment and diagnostic criteria for diseases	**4-5**	**Line 127-170**
		e)	Provide details of ethics committee approval and participant informed consent, if relevant	**11**	**Line 393-395**
5	**Assumptions**		Explicitly state the three core IV assumptions for the main analysis (relevance, independence and exclusion restriction) as well assumptions for any additional or sensitivity analysis	**6**	**Line 202-228**
6	**Statistical methods: main analysis**		Describe statistical methods and statistics used	**5**	**Line 174-200**

		a)	Describe how quantitative variables were handled in the analyses (i.e., scale, units, model)	**6**	**Line 225-228**<br>**Supplementary table 1**
		b)	Describe how genetic variants were handled in the analyses and, if applicable, how their weights were selected	**6**	**Line 225-228**<br>**Supplementary table 1**
		c)	Describe the MR estimator (e.g. two-stage least squares, Wald ratio) and related statistics. Detail the included covariates and, in case of two-sample MR, whether the same covariate set was used for adjustment in the two samples	**5**	**Line 174-200**
		d)	Explain how missing data were addressed	**6**	**Line 203-214**
		e)	If applicable, indicate how multiple testing was addressed	**N/A**	**N/A**
7	**Assessment of assumptions**		Describe any methods or prior knowledge used to assess the assumptions or justify their validity	**6**	**Line 203-214**
8	**Sensitivity analyses and additional analyses**		Describe any sensitivity analyses or additional analyses performed (e.g. comparison of effect estimates from different approaches, independent replication, bias analytic techniques, validation of instruments, simulations)	**5**	**Line 182-188**
9	**Software and pre-registration**				
		a)	Name statistical software and package(s), including version and settings used	**6**	**Line 197-200**
		b)	State whether the study protocol and details were pre-registered (as well as when and where)	**N/A**	**N/A**
	**RESULTS**				
10	**Descriptive data**				
		a)	Report the numbers of individuals at each stage of included studies and reasons for exclusion. Consider use of a flow diagram	**6-7**	**Line 233-238**<br>**Line 256-258**<br>**Line 269-270**
		b)	Report summary statistics for phenotypic exposure(s), outcome(s), and other relevant variables (e.g. means, SDs, proportions)	**N/A**	**Supplementary table 5-9**
		c)	If the data sources include meta-analyses of previous studies, provide the assessments of heterogeneity across these studies	**N/A**	**N/A**
		d)	For two-sample MR:<br>i. Provide justification of the similarity of the genetic variant-exposure associations between the exposure and outcome samples<br>ii. Provide information on the number of individuals who overlap between the exposure and outcome studies	**4-4**	**Line 129-170**<br>**Supplementary table 1**

11	**Main results**				
		a)	Report the associations between genetic variant and exposure, and between genetic variant and outcome, preferably on an interpretable scale	**6-8**	**Line 233-286**<br>**Supplementary table 5-9**
		b)	Report MR estimates of the relationship between exposure and outcome, and the measures of uncertainty from the MR analysis, on an interpretable scale, such as odds ratio or relative risk per SD difference	**6-8**	**Line 233-286**<br>**Supplementary table 5-9**
		c)	If relevant, consider translating estimates of relative risk into absolute risk for a meaningful time period	**N/A**	**N/A**
		d)	Consider plots to visualize results (e.g. forest plot, scatterplot of associations between genetic variants and outcome versus between genetic variants and exposure)	**N/A**	**fig 1-4**
12	**Assessment of assumptions**				
		a)	Report the assessment of the validity of the assumptions	**6-7**	**Line 233-238**<br>**Line 256-258**<br>**Line 269-270**
		b)	Report any additional statistics (e.g., assessments of heterogeneity across genetic variants, such as I^2 , Q statistic or E-value)	**6-8**	**Line 248-253**<br>**Line 264-266**<br>**Line 275-277**<br>**Supplementary table 2-4**
13	**Sensitivity analyses and additional analyses**				
		a)	Report any sensitivity analyses to assess the robustness of the main results to violations of the assumptions	**6-8**	**Line 239-243**<br>**Line 259-263**<br>**Line 211-274**<br>**Supplementary table 5-9**
		b)	Report results from other sensitivity analyses or additional analyses	**6-8**	**Line 239-243**<br>**Line 259-263**<br>**Line 211-274**<br>**Supplementary table 5-9**
		c)	Report any assessment of direction of causal relationship (e.g., bidirectional MR)	**7**	**Line 268-277**
		d)	When relevant, report and compare with estimates from non-MR analyses	**N/A**	**N/A**
		**e)**	Consider additional plots to visualize results (e.g., leave-one-out analyses)	**N/A**	**Fig 1-4**

	**DISCUSSION**				
14	**Key results**		Summarize key results with reference to study objectives	**8**	**Line 290-299**
15	**Limitations**		Discuss limitations of the study, taking into account the validity of the IV assumptions, other sources of potential bias, and imprecision. Discuss both direction and magnitude of any potential bias and any efforts to address them	**9**	**Line 362-267**
16	**Interpretation**				
		a)	Meaning: Give a cautious overall interpretation of results in the context of their limitations and in comparison with other studies	**8-9**	**Line 301-351**
		b)	Mechanism: Discuss underlying biological mechanisms that could drive a potential causal relationship between the investigated exposure and the outcome, and whether the gene-environment equivalence assumption is reasonable. Use causal language carefully, clarifying that IV estimates may provide causal effects only under certain assumptions	**8-9**	**Line 342-251**
		c)	Clinical relevance: Discuss whether the results have clinical or public policy relevance, and to what extent they inform effect sizes of possible interventions	**10**	**Line 372-374**
17	**Generalizability**		Discuss the generalizability of the study results (a) to other populations, (b) across other exposure periods/timings, and (c) across other levels of exposure	**9**	**Line 362-363**
	**OTHER INFORMATION**				
18	**Funding**		Describe sources of funding and the role of funders in the present study and, if applicable, sources of funding for the databases and original study or studies on which the present study is based	**11**	**Line 382-383**
19	**Data and data sharing**		Provide the data used to perform all analyses or report where and how the data can be accessed, and reference these sources in the article. Provide the statistical code needed to reproduce the results in the article, or report whether the code is publicly accessible and if so, where	**4-5**	**Line 128-174**
20	**Conflicts of Interest**		All authors should declare all potential conflicts of interest	**11**	**Line 386**

[i] Skrivankova VW, Richmond RC, Woolf BAR, Yarmolinsky J, Davies NM, Swanson SA, VanderWeele TJ, Higgins JPT, Timpson NJ, Dimou N, Langenberg C, Golub RM, Loder EW, Gallo V, Tybjaerg-Hansen A, Davey Smith G, Egger M, Richards JB. Strengthening the Reporting of Observational Studies in Epidemiology Using Mendelian Randomization: The STROBE-MR Statement. JAMA. 2021 Oct 26;326(16):1614-1621. doi: 10.1001/jama.2021.18236. PMID: 34698778.

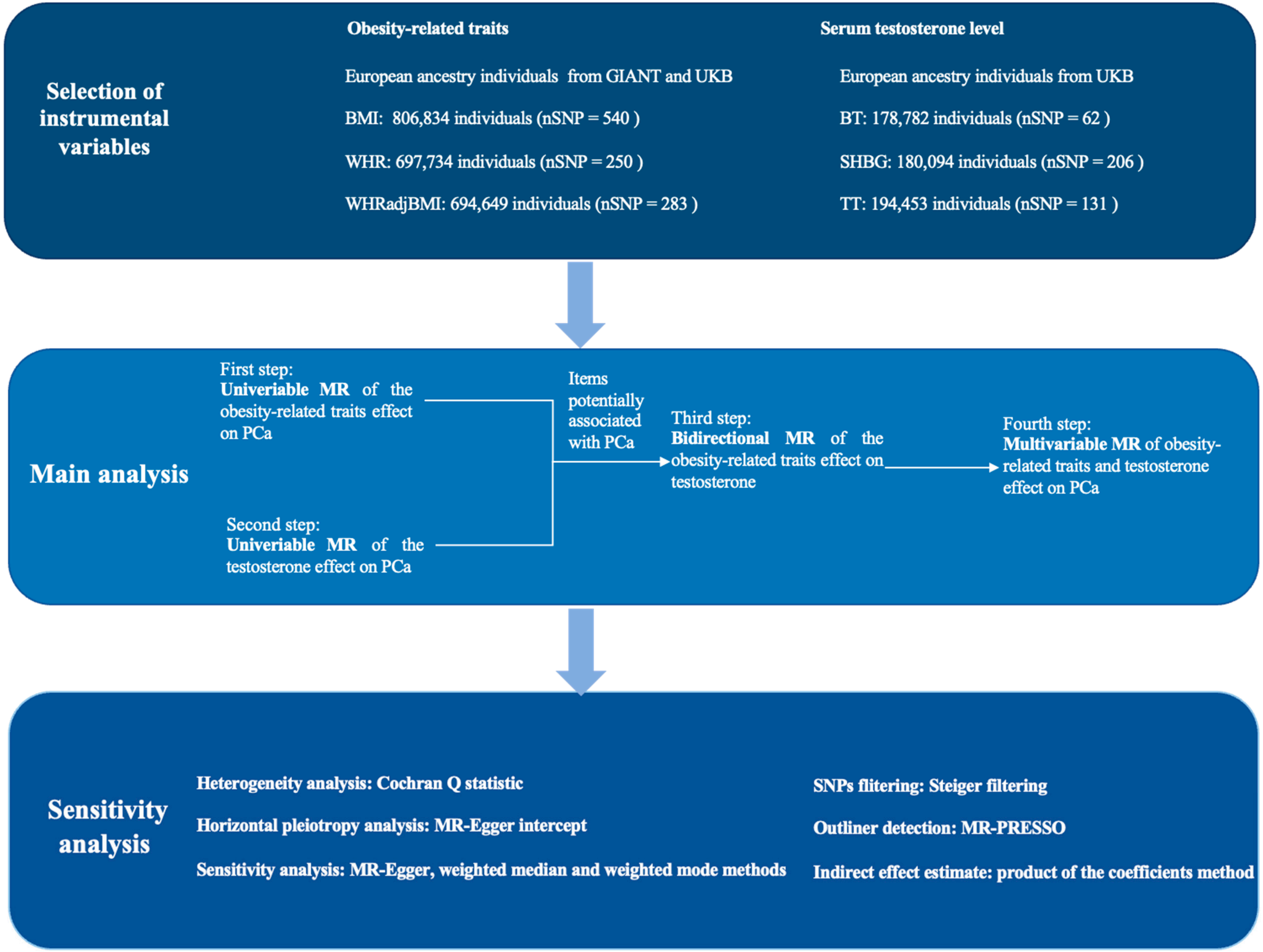

Supplementary Figure S1: Overview of the study design.

MR, Mendelian randomization; PCa, prostate cancer; BMI, Body Mass Index; WHR, waist-to-hip ratio; WHRadjBMI, waist-to-hip ratio adjusted for body mass index; SNP, single nucleotide polymorphism; UKB, UK biobank; PRACTICAL, Prostate Cancer Association Group to Investigate Cancer-Associated Alterations in the Genome; BT, Bioavailable testosterone; TT, total testosterone; SHBG, sex hormone-binding globulin
